# MicroRNA-139-3p regulates osteoblast differentiation and apoptosis by targeting ELK1 and interacting with long noncoding RNA ODSM

**DOI:** 10.1038/s41419-018-1153-1

**Published:** 2018-10-31

**Authors:** Yixuan Wang, Ke Wang, Zebing Hu, Hua Zhou, Lijun Zhang, Han Wang, Gaozhi Li, Shu Zhang, Xinsheng Cao, Fei Shi

**Affiliations:** The Key Laboratory of Aerospace Medicine, Ministry of Education, Air Force Medical University, Xi’an, Shaanxi 710032 China

## Abstract

Recent studies have confirmed that microRNAs and lncRNAs can affect bone cell differentiation and bone formation. In this study, miR-139-3p was upregulated in the femurs of hindlimb unloading mice and MC3T3-E1 cells under simulated microgravity; this effect was related to osteoblast differentiation and apoptosis. Silencing miR-139-3p attenuated the suppression of differentiation and the promotion of MC3T3-E1 cell apoptosis induced by simulated microgravity. ELK1 is a target of miR-139-3p and is essential for miR-139-3p to regulate osteoblast differentiation and apoptosis. An osteoblast differentiation-related lncRNA that could interact with miR-139-3p (lncRNA ODSM) was identified in MC3T3-E1 cells under simulated microgravity. Further investigations demonstrated that lncRNA ODSM could promote MC3T3-E1 cell differentiation. Therefore, this research was the first to reveal the critical role of the lncRNA ODSM/miR-139-3p/ELK1 pathway in osteoblasts, and these findings suggest the potential value of miR-139-3p in osteoporosis diagnosis and therapy.

## Introduction

Osteoporosis is related to several risk factors, including hormone fluctuation, nutrition, and inflammatory and mechanical stress^1–3^. Bone loss induced by microgravity is similar to osteoporosis in that bone mass is remarkably decreased, and the microarchitectures of the bone are markedly altered. Bone loss induced by microgravity is a critical phenomenon occurring in humans; this process is the most serious threat to astronauts’ health during spaceflight^3–5^. Because of the infrequency and tremendous costs of space flights, most studies have been performed on earth using simulated microgravity. Hindlimb unloading animal models and human bed-rest studies are most commonly conducted using in vivo models, which mimic the lack of weight-bearing loads on bones and cephalic fluid shifts in spaceflight^6,7^. In vitro simulators of microgravity utilize mainly clinostat, random positioning machines or rotary wall vessels, which are used to study cell responses to conditions lacking weight-bearing forces^8–10^.

Studies have shown that reduced bone formation is the primary characteristic of bone loss during spaceflight^11–13^. Bone formation is regulated by biological and mechanical factors, such as transcription factors and signaling pathways, at multiple regulatory levels^14–18^. miRNAs, which are small noncoding RNAs, have been demonstrated to regulate gene expression at the posttranscriptional level^19–21^. Recent studies have indicated that miRNAs, which can regulate bone formation at all stages, are associated with osteoporosis and other bone diseases^22,23^. Additionally, some miRNAs were found to be sensitive to microgravity and have a marked effect on osteoblast functions. Our previous studies showed that miR-132-3p and miR-103 were upregulated in pre-osteoblast MC3T3-E1 cells to inhibit osteoblast proliferation and differentiation under simulated microgravity^24,25^. In addition, miR-33-5p, which is negatively induced by mechanical force, can promote osteoblast differentiation in MC3T3-E1 cells under simulated microgravity^26^. Although several miRNAs have been demonstrated to regulate the proliferation and differentiation of osteoblasts, whether miRNAs could regulate osteoblast apoptosis under simulated microgravity and the relationship between the majority of miRNAs and microgravity-caused bone loss remain to be explored.

Long noncoding RNAs (lncRNAs), which have more than 200 nucleotides and no coding potential, have been shown to act as competitive endogenous RNAs that regulate the expression and activity of miRNAs^27–29^. Recent studies have demonstrated that lncRNAs are involved in osteogenic differentiation. For example, in periodontal mesenchymal stem cells, lncRNA-POIR interacted with miR-182 to upregulate the function of osteogenic differentiation^30^. Knocking down lncRNA MEG3 inhibited the ability of mesenchymal stem cells to differentiate into osteoblasts^31^. However, the role and mechanism of lncRNAs in the regulation of osteoblast functions in a microgravity environment are still largely unknown.

Our present study reports for the first time that miR-139-3p was upregulated in the femurs of hindlimb unloading mice and MC3T3-E1 cells under simulated microgravity; this effect could suppress osteoblast differentiation and promote osteoblast apoptosis. ELK1, an ETS transcription factor, has been reported as a target of miR-139-3p and is essential for miR-139-3p to regulate osteoblast functions. Further studies identified that the lncRNA NONMMUT002009 (lncRNA ODSM), which is an osteoblast differentiation-related lncRNA, could interact with miR-139-3p and promote osteoblast differentiation in MC3T3-E1 cells under simulated microgravity. Our studies determined the molecular function of the lncRNA ODSM/miR-139-3p/ELK1 pathway in osteoblasts and established the potential value of miR-139-3p in preventative treatment for disuse osteoporosis.

## Results

### MiR-139-3p is upregulated in the femurs of hindlimb unloading mice and MC3T3-E1 cells under simulated microgravity

**Fig. 1:**
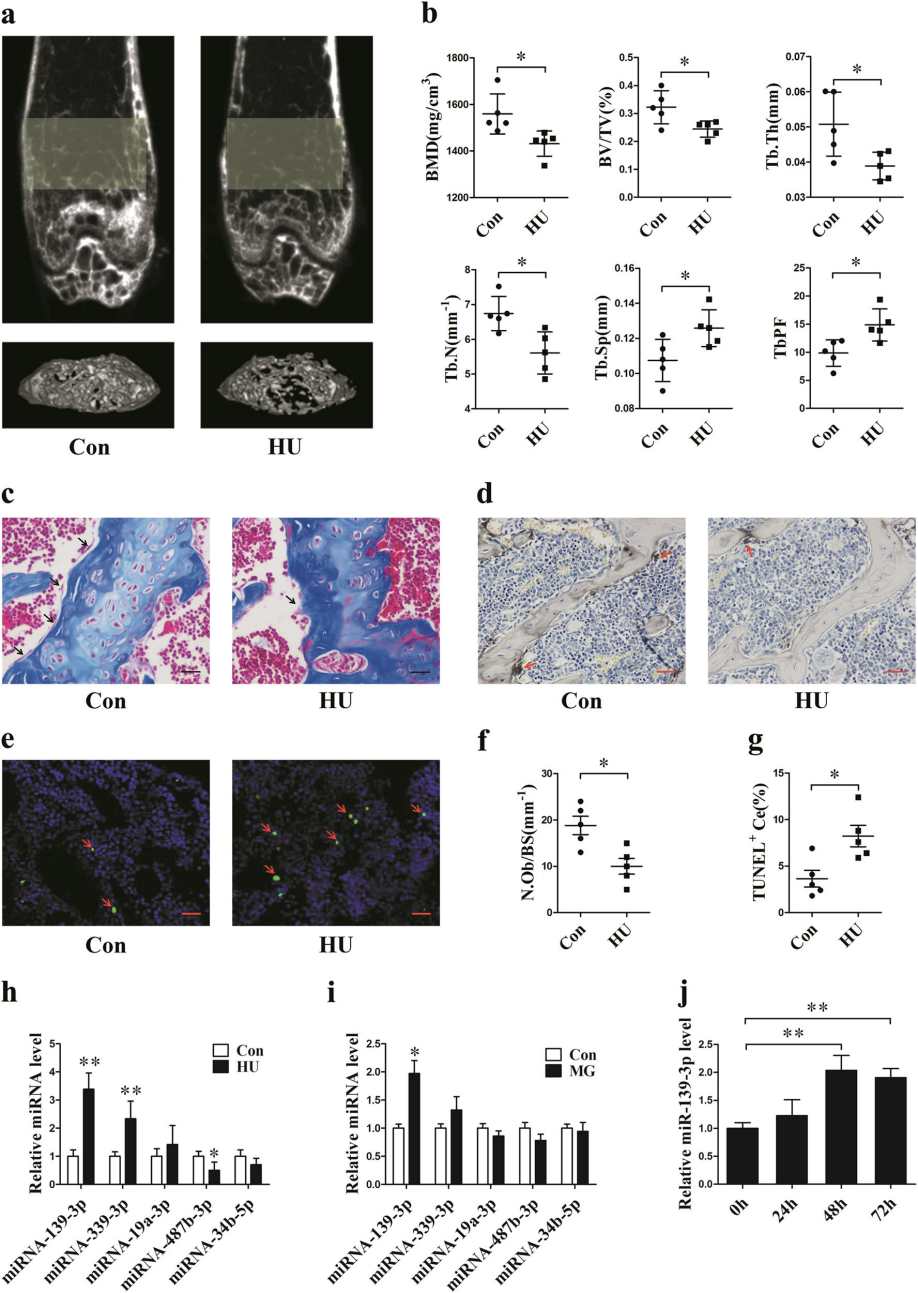
MiR-139-3p is upregulated in the femurs of hindlimb unloading mice and MC3T3-E1 cells under simulated microgravity. **a** Representative images determined by μCT examination for the trabecular architecture of the distal femurs of mice from each group (*N* = 5). **b** μCT analysis of the ROI region of the distal femurs of mice from each group. The three-dimensional indices were bone mineral density (BMD), relative bone volume (BV/TV), trabecular bone thickness (Tb.Th), trabecular bone number (Tb.N), trabecular bone separation (Tb.Sp), and trabecular bone pattern factor (TbPF) (*N* = 5). **c** Representative images of Masson staining of the distal femurs of mice from the CON and HU groups (*N* = 5). Scale bar, 50 µm. The arrows indicate osteoids. **d** Representative staining images of ALP in the distal femurs. Scale bar, 50 µm (*N* = 5). The arrows show osteoblasts. **e** Representative images of TUNEL staining of the distal femurs of mice from the CON and HU groups (*N* = 5). Scale bar, 25 µm. The arrows show apoptotic cells. **f** Statistical analysis of the number of positive osteoblasts per bone surface of the femurs (N.Ob/BS) (*N* = 5). **g** Statistical analysis of TUNEL-positive cell percentages (*N* = 3). **h** qRT-PCR analysis of miR-139-3p, miR-339-3p, miR-19a-3p, miR-487b-3p, and miR-34b-5p levels selected from the array data of the distal femurs of mice from each group (*N* = 5). **i** qRT-PCR analysis of candidate miRNA levels under clinorotation conditions for 48 h (*N* = 3). **j** qRT-PCR analysis of the miR-139-3p expression pattern under clinorotation conditions for 72 h (*N* = 3). *P *<* 0.05, **P *<* 0.01 vs. control

To explore the expression and significance of miRNAs in mouse osteoblasts under simulated microgravity, hindlimb unloaded (HU) mice and cells under clinorotation conditions were selected as models. After 21 days of hindlimb unloading, the MicroCT analysis showed remarkable decreases in the bone mineral density (BMD), relative bone volume (BV/TV), trabecular bone thickness (Tb.Th) and trabecular bone number (Tb.N), with significant increases in trabecular bone separation (Tb.Sp) and trabecular bone pattern factor (TbPF) in the HU group compared with those in the Con group (Fig. 1a, b). Furthermore, Masson staining indicated that compared to Con mice, HU mice showed less osteoid staining in the distal femur (Fig. 1c). The ALP staining results showed that ALP-positive osteoblast areas were also significantly decreased in the femurs of HU mice (Fig. 1d–f). In addition, the proportions of TUNEL-positive apoptotic cells were significantly higher in the distal femurs of HU mice than in those of Con mice (Fig. 1e–g).

According to the microarray data of our previous miRNA study, miRNA-139-3p, miRNA-339-3p, miRNA-19a-3p, miRNA-487b-3p, and miRNA-34b-5p were selected as candidate miRNAs^24^. Next, we confirmed the microarray results by qRT-PCR, and the expression levels of miR-139-3p, miR-339-3p, and miR-487b-3p were changed remarkably during bone loss (Fig. 1h). To explore the relationship between the differentially expressed miRNAs and osteoblast differentiation under simulated microgravity (MG), mouse pre-osteoblast MC3T3-E1 cells were cultured under clinorotation conditions for 48 h. Among the differentially expressed miRNAs, miR-139-3p was markedly upregulated in the MG group (Fig. 1i); this change was similar to that in vivo. A time-dependent experiment showed that miR-139-3p expression was continuously upregulated and peaked at 48 h under clinorotation conditions (Fig. 1j).

### MiR-139-3p inhibits osteoblast differentiation and promotes apoptosis in MC3T3-E1 cells

**Fig. 2:**
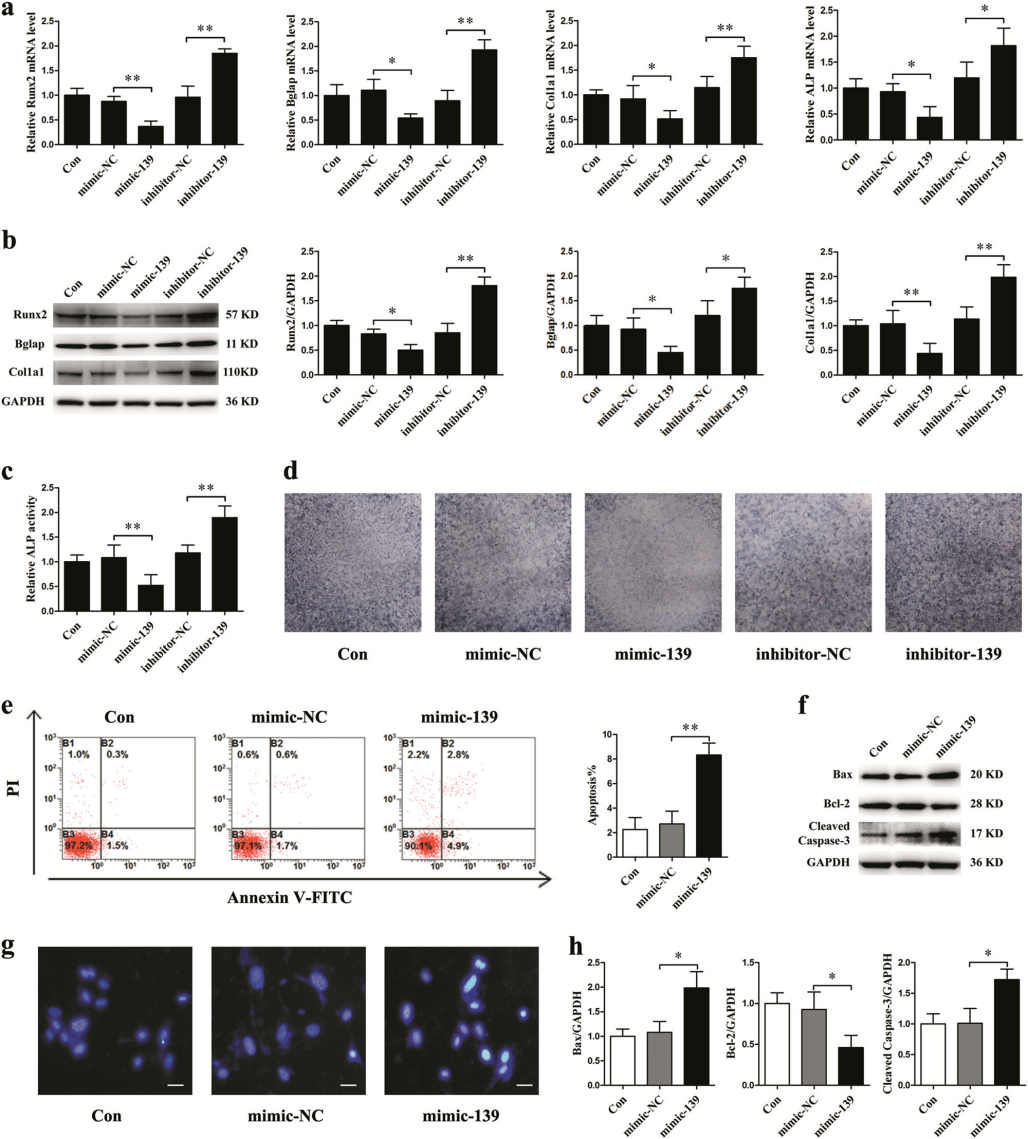
MiR-139-3p inhibits osteoblast differentiation and promotes osteoblast apoptosis. miR-139-3p mimic, inhibitor, and miRNA negative control were transfected into MC3T3-E1 cells. **a** qRT-PCR analysis of osteoblast marker gene (Runx2, Bglap, Col1a1, and ALP normalized to GAPDH) mRNA levels in osteoblasts (*N* = 3). **b** Western blotting analyses of Runx2, Bglap, and Col1a1 protein expression levels in osteoblasts (*N* = 3). **c** ALP activity analysis of osteoblasts at 48 h (*N* = 3). **d** Representative staining images of ALP in osteoblasts (*N* = 3). **e** Flow cytometry analysis of apoptosis in osteoblasts stained with Annexin V-FITC/PI (*N* = 3). **f**, **h** Protein levels of Bax, Bcl-2, and cleaved caspase-3 in osteoblasts (*N* = 3). **g** Representative images of Hoechst 33258 staining in osteoblasts (*N* = 3). Scale bar, 50 µm. *P *<* 0.05, **P *<* 0.01 vs. control

To explore the biological effects of miR-139-3p on osteogenic differentiation in vitro, mimic-139 and inhibitor-139 were used to change the intracellular levels of miR-139-3p in MC3T3-E1 cells. The results demonstrated that mimic-139 significantly decreased the osteogenic gene expression of Runx2, Bglap, Col1a1, and ALP, whereas inhibitor-139 increased these gene expression levels in the cells (Fig. 2a). The protein expression levels of Runx2, Bglap, and Col1a1 were remarkably decreased in the group treated with mimic-139 and conversely enhanced in the group treated with inhibitor-139 (Fig. 2b). ALP activity and ALP staining were significantly decreased in the group treated with mimic-139, whereas they were enhanced in the inhibitor-139 group (Fig. 2c, d). Moreover, the ratio of apoptotic cells detected by flow cytometry was significantly increased in the mimic-139 group (Fig. 2e). Levels of the apoptosis-associated proteins Bax and cleaved caspase-3 were remarkably increased in the mimic-139 group, whereas Bcl-2 protein levels were markedly decreased (Fig. 2f–h). To evaluate the apoptotic status of MC3T3-E1 cells, Hoechst 33258 staining was used. Blue apoptotic nuclei were observed in mimic-139-treated cells (Fig. 2g).

### MiR-139-3p silencing partly alleviates the alterations in differentiation and apoptosis in MC3T3-E1 cells under simulated microgravity

**Fig. 3:**
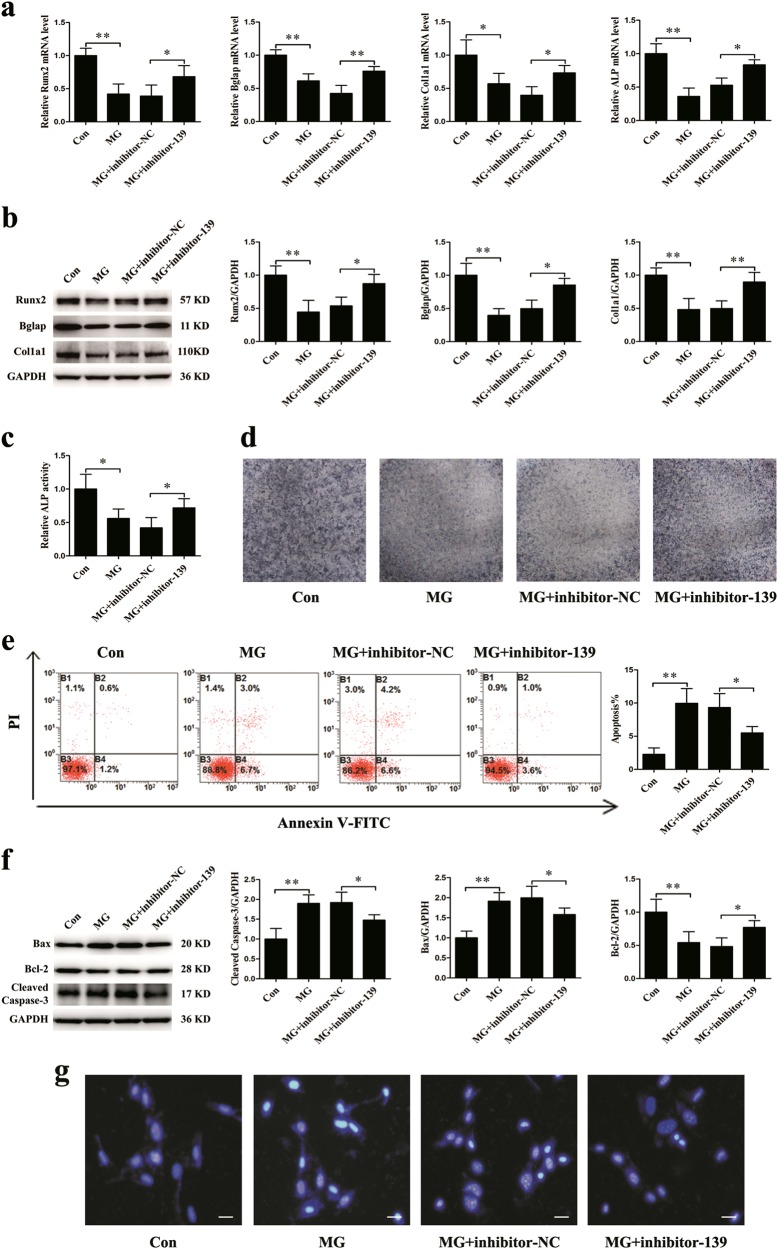
Silencing miR-139-3p partly alleviates the alterations in differentiation and apoptosis in MC3T3-E1 cells under simulated microgravity conditions for 48 h. **a** mRNA expression levels of osteoblast marker genes (Runx2, Bglap, Col1a1, and ALP normalized to GAPDH) in osteoblasts treated with inhibitor-139 or the corresponding control (*N* = 3). **b** Protein levels of Runx2, Bglap, and Col1a1 in osteoblasts (*N* = 3). **c** ALP activity analysis in osteoblasts at 48 h (*N* = 3). **d** Representative staining images for ALP in osteoblasts (*N* = 3). **e** Flow cytometry analysis of apoptosis in osteoblasts stained with Annexin V-FITC/PI (*N* = 3). **f** Protein levels of Bax, Bcl-2, and cleaved caspase-3 in osteoblasts (*N* = 3). **g** Representative images of Hoechst 33258 staining in osteoblasts (*N* = 3). Scale bar, 50 µm. *P *<* 0.05, **P *<* 0.01 vs. control

It is well-known that simulated microgravity can suppress osteogenic differentiation in osteoblasts^13,24,26^. To explore whether miR-139-3p can affect osteoblast differentiation under simulated microgravity, MC3T3-E1 cells were transfected with inhibitor-139 for 12 h and then cultured in a simulated microgravity environment for 48 h. The mRNA and protein expression levels of the osteogenic markers Runx2, Bglap, and Col1a1 were markedly increased in the inhibitor-139 group. Moreover, inhibitor-139 markedly promoted the gene expression and protein activity of ALP (Fig. 3a–d). These data demonstrate that knocking down endogenous miR-139-3p expression partly alleviates the inhibition of osteoblast differentiation in MC3T3-E1 cells under simulated microgravity. On the other hand, the proportion of apoptotic cells was remarkably decreased in the inhibitor-139 group (Fig. 3e). Levels of the apoptosis-associated proteins Bax and cleaved caspase-3 were significantly decreased in the inhibitor-139 group, whereas Bcl-2 protein levels were markedly increased (Fig. 3f). The apoptotic status of MC3T3-E1 cells was shown by Hoechst 33258 staining. The number of blue apoptotic nuclei was decreased in the inhibitor-139 group under a simulated microgravity environment (Fig. 3g).

### MiR-139-3p directly targets ELK1 in MC3T3-E1 cells

**Fig. 4:**
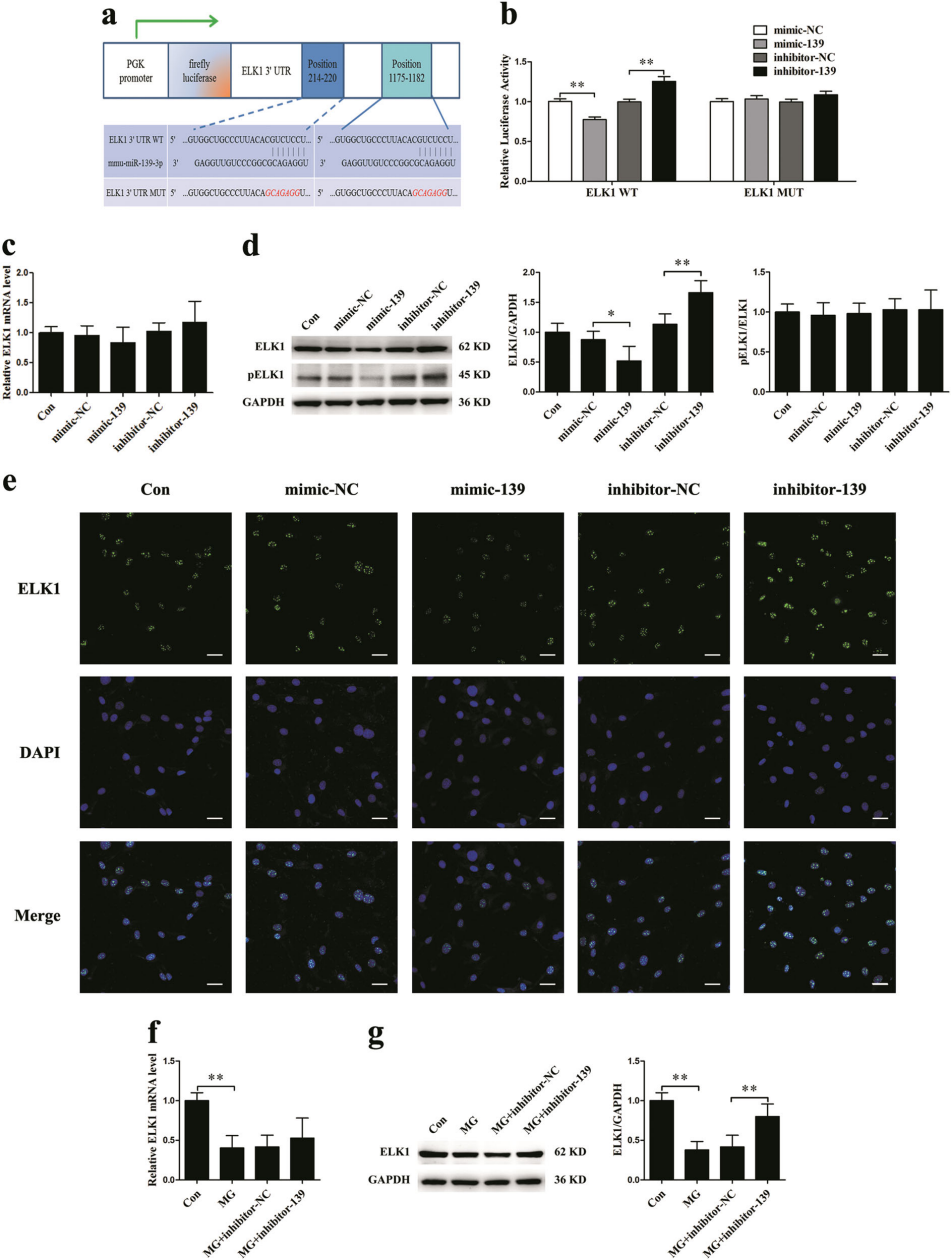
MiR-139-3p directly targets ELK1 in MC3T3-E1 cells, and ELK1 is sensitive to simulated microgravity. **a** Schematic representation of the luciferase reporter containing ELK1 3′UTR WT or ELK1 3′UTR MUT. **b** The luciferase activity of the ELK1 WT and ELK1 MUT reporter in 293T cells treated with mimic-139, inhibitor-139 or the corresponding controls for 24 h (*N* = 3). **c** mRNA expression of ELK1 in osteoblasts treated with mimic-139, inhibitor-139 or the corresponding controls for 48 h (*N* = 3). **d** Protein levels of ELK1 and pELK1 in osteoblasts (*N* = 3). **e** Immunostaining analysis of the expression levels of ELK1 in osteoblasts (*N* = 3). Scale bar, 50 µm. **f** mRNA expression of ELK1 in osteoblasts under clinorotation conditions for 48 h (*N* = 3). **g** Protein levels of ELK1 in osteoblasts treated with inhibitor-139 or the corresponding control under clinorotation conditions for 48 h (*N* = 3). *P *<* 0.05, **P *<* 0.01 vs. control

To explore the regulatory mechanism of miR-139-3p in osteoblast differentiation, TargetScan, miRanda, and miRDB were used to analyze potential target genes (Supplementary Table 2). Based on these analyses, we found that ELK1, which has been reported to be related to osteoblast differentiation, was the most promising candidate^32–35^. To confirm the bioinformatics prediction results, we constructed luciferase reporters containing either the wild-type ELK1 3′UTR sequence (WT) or an ELK1 3′UTR mutant sequence of the miR-139-3p binding site (MUT) (Fig. 4a). Next, luciferase reporter assays demonstrated that only ELK1 3′UTR WT luciferase reporter activity was significantly reduced by mimic-139 and markedly increased by inhibitor-139; the changes in ELK1 3′UTR MUT reporter activity were not statistically significant (Fig. 4b). These results indicate that miR-139-3p directly interferes with ELK1 expression. Furthermore, qRT-PCR results demonstrated that miR-139-3p does not significantly influence ELK1 gene expression in MC3T3-E1 cells (Fig. 4c). However, western blotting analyses showed that the protein translation of ELK1 was suppressed by miR-139-3p, and this effect had little influence on ELK1 phosphorylation (Fig. 4d). Indirect immunofluorescence assays showed that mimic-139 transfection was associated with decreased ELK1 protein expression levels, whereas inhibitor-139 transfection yielded the opposite results (Fig. 4e).

To investigate ELK1 expression in osteoblasts under simulated microgravity, MC3T3-E1 cells were cultured under clinorotation conditions for 48 h. The gene and protein expression levels of ELK1 were markedly decreased under simulated microgravity. Moreover, inhibitor-139 could attenuate only the downregulated ELK1 protein levels but not the mRNA levels (Fig. 4f, g).

### ELK1 promotes osteoblast differentiation and inhibits osteoblast apoptosis

**Fig. 5:**
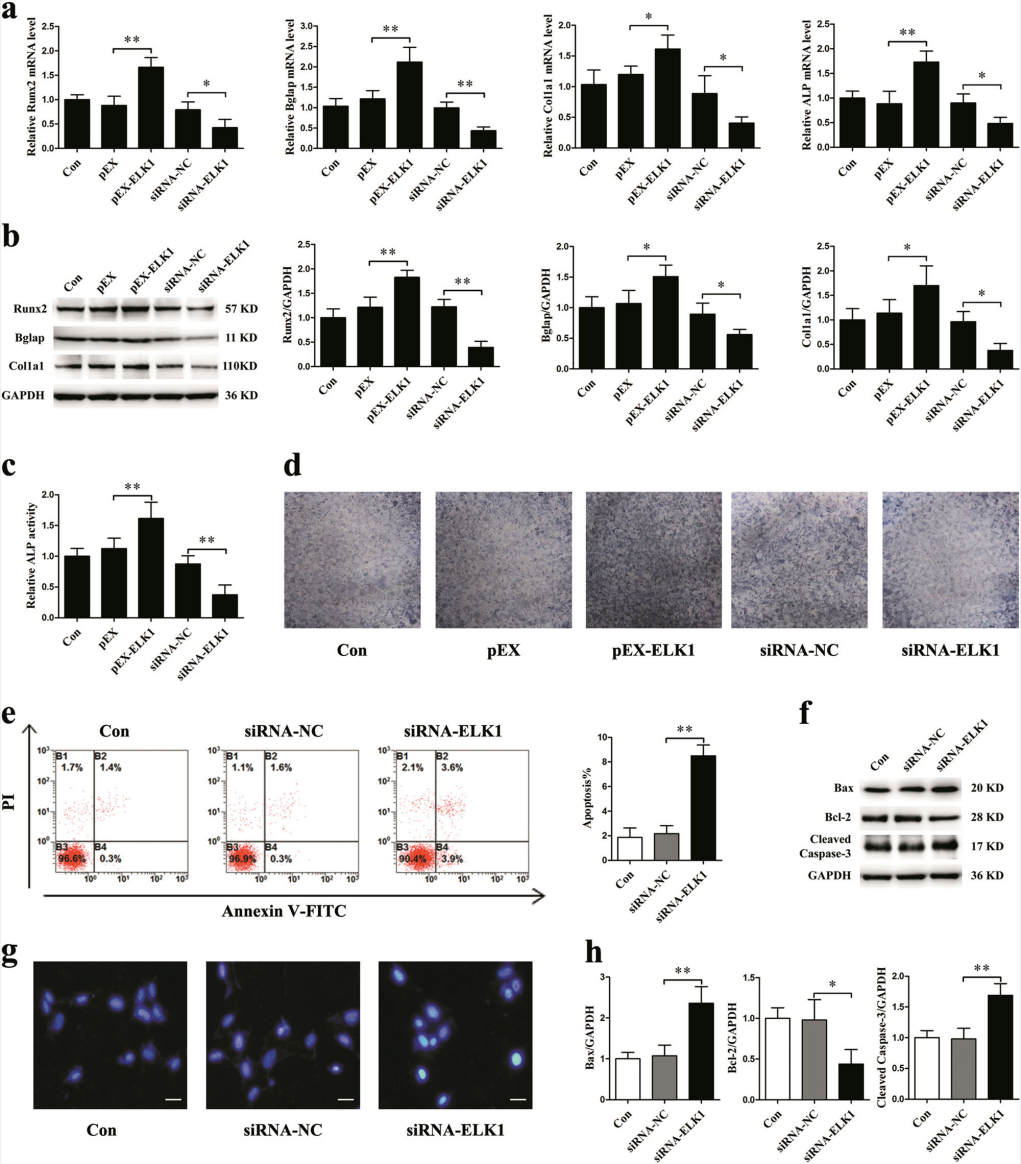
ELK1 promotes osteoblast differentiation and inhibits osteoblast apoptosis. pEX-ELK1, siRNA-ELK1, and their negative controls were transfected into MC3T3-E1 cells. **a** qRT-PCR analysis of osteoblast marker gene (Runx2, Bglap, Col1a1, and ALP normalized to GAPDH) mRNA levels in osteoblasts (*N* = 3). **b** Western blotting analyses of Runx2, Bglap, and Col1a1 protein expression in osteoblasts (*N* = 3). **c** ALP activity analysis in osteoblasts at 48 h (*N* = 3) (*N* = 3). **d** Representative images of ALP staining in osteoblasts (*N* = 3). **e** Flow cytometry analysis of apoptosis in osteoblasts stained with Annexin V-FITC/PI (*N* = 3). **f**, **h** Protein levels of Bax, Bcl-2, and cleaved caspase-3 in osteoblasts (*N* = 3). **g** Representative images of Hoechst 33258 staining in osteoblasts (*N* = 3). Scale bar, 50 µm. *P *<* 0.05, **P *<* 0.01 vs. control

To further explore the role of ELK1, we used an overexpression vector (pEX-ELK1) to overexpress ELK1 and RNA interference to knockdown ELK1 in MC3T3-E1 cells. Consequently, pEX-ELK1 transfection significantly upregulated Runx2, Bglap, Col1a1, and ALP gene expression, whereas siRNA-ELK1 transfection considerably decreased the mRNA expression levels of these osteogenic genes (Fig. 5a). The changes in Runx2, Bglap and Col1a1 protein levels, ALP activity, and ALP expression also showed similar trends (Fig. 5b–d). In the siRNA-ELK1 group, the ratio of apoptotic osteoblasts was remarkably increased (Fig. 5e). Moreover, Bax and cleaved caspase-3 protein expression levels were increased, whereas Bcl-2 was downregulated in the siRNA-ELK1 group (Fig. 5f–h). Blue apoptotic nuclei stained by Hoechst 33258 were observed in siRNA-ELK1-treated cells (Fig. 5g).

### ELK1 is essential for miR-139-3p to inhibit osteoblast differentiation and promote osteoblast apoptosis

**Fig. 6:**
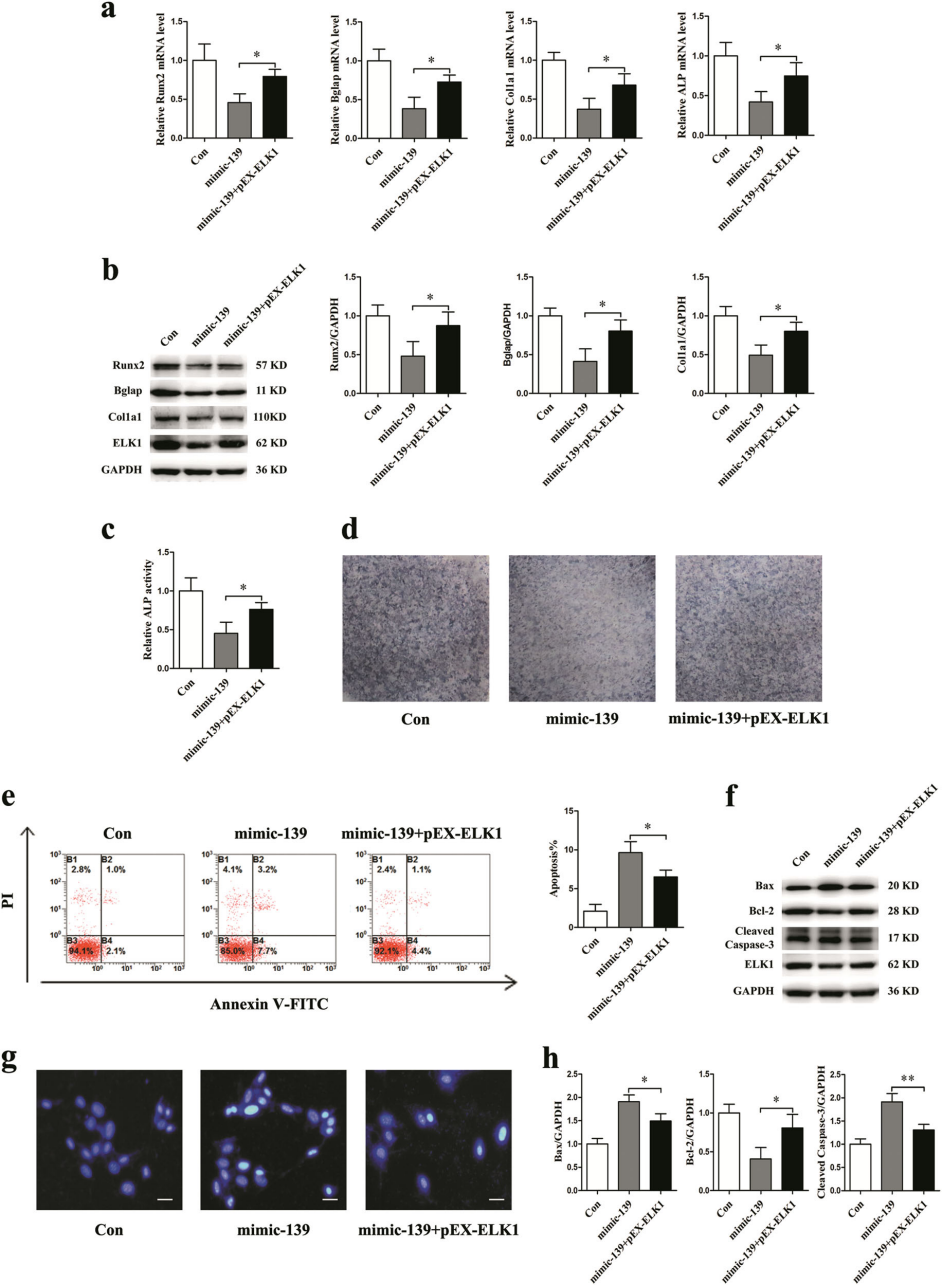
ELK1 is essential for miR-139-3p to inhibit osteoblast differentiation and promote osteoblast apoptosis. Mimic-139 and pEX-ELK1 were co-transfected into MC3T3-E1 cells. **a** qRT-PCR analysis of osteoblast marker genes (Runx2, Bglap, Col1a1, and ALP normalized to GAPDH) mRNA levels in osteoblasts (*N* = 3). **b** Western blotting analyses of Runx2, Bglap, and Col1a1 protein expression in osteoblasts (*N* = 3). **c** ALP activity analysis in osteoblasts at 48 h (*N* = 3). **d** Representative images of ALP staining in osteoblasts (*N* = 3). **e** Flow cytometry analysis of apoptosis in osteoblasts stained with Annexin V-FITC/PI (*N* = 3). **f**, **h** Protein levels of Bax, Bcl-2, and cleaved caspase-3 in osteoblasts (*N* = 3). **g** Representative images of Hoechst 33258 staining in osteoblasts (*N* = 3). Scale bar, 50 µm. *P *<* 0.05, **P *<* 0.01 vs. control

To confirm that the promotion of apoptosis and suppression of differentiation by miR-139-3p depends on ELK1 in osteoblasts, mimic-139, and pEX-ELK1 or its negative control were co-transfected into MC3T3-E1 cells. pEX-ELK1 significantly blocked the mimic-139-induced decrease in Runx2, Bglap, Col1a1, and ALP mRNA levels (Fig. 6a). The reductions in Runx2, Bglap, and Col1a1 protein levels were significantly blocked by pEX-ELK1 (Fig. 6b). ALP activity assays and ALP staining showed similar changes (Fig. 6c, d). Following co-transfection with mimic-139 and pEX-ELK1, the ratio of apoptotic osteoblasts was markedly decreased in the pEX-ELK1 group (Fig. 6e). pEX-ELK1 significantly attenuated the increased Bax and cleaved caspase-3 protein levels and decreased Bcl-2 protein levels (Fig. 6f–h). There were fewer blue apoptotic nuclei stained by Hoechst 33258 in the pEX-ELK1 and mimic-139 co-transfection group than in the mimic-139 group (Fig. 6g).

### LncRNA ODSM and miR-139-3p interact with and repress each other

**Fig. 7:**
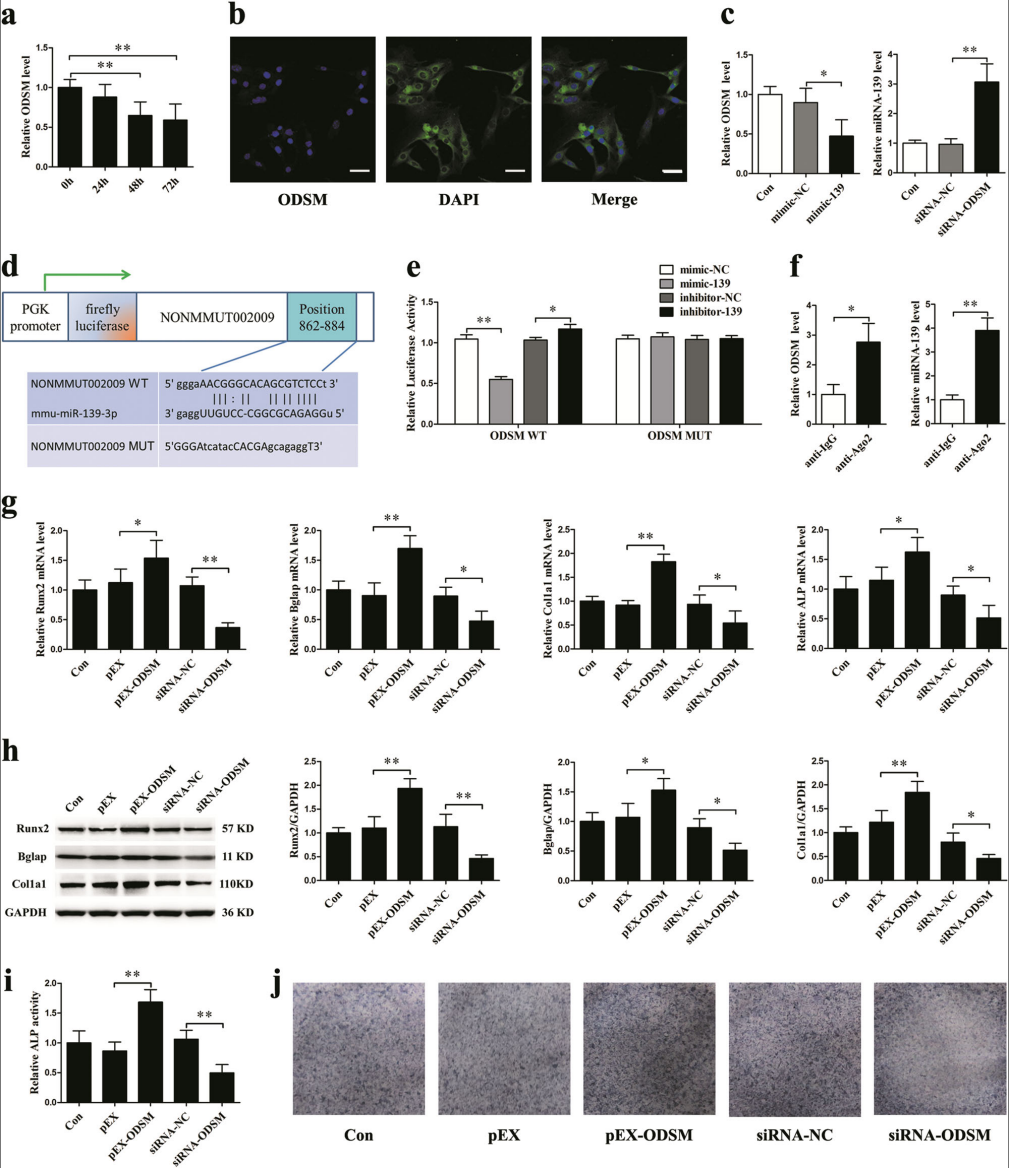
LncRNA ODSM and miR-139-3p interact with and repress each other, while lncRNA ODSM promotes osteoblast differentiation. **a** qRT-PCR analysis of lncRNA ODSM expression patterns in osteoblasts under clinorotation conditions for 72 h (*N* = 3). **b** The subcellular localization of lncRNA ODSM in MC3T3-E1 cells determined by RNA fluorescence in situ hybridization (*N* = 3). Scale bar, 50 µm. **c** qRT-PCR analysis of lncRNA ODSM mRNA levels in osteoblasts treated with mimic-139 or its negative control and miR-139 expression levels in osteoblasts treated with siRNA-ODSM or its negative control (*N* = 3). **d** Schematic representation of the luciferase reporter containing the ODSM WT or the ODSM MUT sequence. **e** The luciferase activity of the ODSM WT or ODSM MUT reporter in 293T cells treated with miR-139-3p mimic, inhibitor or the corresponding controls for 24 h (*N* = 3). **f** Ago2 immunoprecipitation was performed using normal mouse IgG as a control, and the expression levels of lncRNA ODSM and miR-139-3p were detected using qRT-PCR in osteoblasts (*N* = 3). **g** Osteoblasts were transfected with pEX-ODSM, siRNA-ODSM or their negative controls. Osteoblast marker gene (Runx2, Bglap, Col1a1, and ALP normalized to GAPDH) mRNA levels were analyzed by qRT-PCR (*N* = 3). **h** Western blotting analyses of Runx2, Bglap, and Col1a1 protein expression in osteoblasts (*N* = 3). **i** ALP activity analysis in osteoblasts at 48 h (*N* = 3). **j** Representative images of ALP staining in osteoblasts (*N* = 3). *P *<* 0.05, **P *<* 0.01 vs. control

A class of lncRNAs that act as competing endogenous RNAs has been reported to interact with miRNAs to protect mRNAs. To determine whether miR-139-3p is regulated by lncRNA, the miRanda database and lncRNA microarray data from our previous study were used to predict potential targets from lncRNAs that were downregulated under simulated microgravity (Supplementary Table 2)^36^. LncRNA ODSM, which was the most promising candidate among the predicted genes, was significantly downregulated in MC3T3-E1 cells under clinorotation conditions (Fig. 7a). Further research confirmed that lncRNA ODSM was located in the cytoplasm and nuclei of cells (Fig. 7b). Mimic-139 significantly decreased lncRNA ODSM expression levels in MC3T3-E1 cells, and silencing lncRNA ODSM significantly increased miR-139-3p expression levels (Fig. 7c). To investigate whether lncRNA ODSM binds directly to miR-139-3p, we constructed luciferase reporters containing lncRNA ODSM wild type (WT) or mutated miR-139-3p binding sites. The luciferase reporter assays demonstrated that mimic-139 significantly decreased and inhibitor-139 increased the luciferase activities of the WT reporter vector but not the mutant reporter vector (Fig. 7d, e). For further confirmation, we conducted an AGO2 immunoprecipitation (RIP) assay. qPCR results showed that the expression levels of lncRNA ODSM and miR-139-3p were markedly higher in the anti-Ago2 group than in the anti-normal IgG group (Fig. 7f).

### LncRNA ODSM promotes osteoblast differentiation

To investigate whether lncRNA ODSM regulates osteogenic differentiation in MC3T3-E1 cells, an overexpression vector and RNA interference were used to examine the effect of lncRNA ODSM. Overexpressing lncRNA ODSM significantly increased the mRNA levels of the osteogenic genes Runx2, Bglap, Col1a1, and ALP, whereas knocking down lncRNA ODSM significantly decreased mRNA levels of these osteogenic genes (Fig. 7g). The protein levels of these osteogenic genes and the ALP activity assay and ALP staining results showed similar changes (Fig. 7h–j).

To investigate whether lncRNA ODSM regulates ELK1 expression in MC3T3-E1 cells, we conducted western blotting analyses. The results showed that overexpressing lncRNA ODSM significantly increased ELK1 protein expression, whereas knocking down lncRNA ODSM significantly decreased ELK1 protein level (Supplementary Fig. 1a). Furthermore, MC3T3-E1 cells were co-transfected with pEX-ODSM and mimic-139 or its negative control for 12 h and then cultured in a simulated microgravity environment for 48 h. Western blotting analyses showed that overexpression of miR-139-3p partly reversed ELK1 level induced by lncRNA ODSM under simulated microgravity (Supplementary Fig. 1b).

## Discussion

An increasing number of studies have confirmed that miRNAs can regulate various cellular processes, such as proliferation, differentiation, and apoptosis. This research was the first to demonstrate that miR-139-3p inhibits osteoblast differentiation and promotes osteoblast apoptosis. miR-139-3p overexpression inhibited differentiation and promoted apoptosis in osteoblasts, especially under clinorotation conditions. These data suggest that miR-139-3p might be a promising treatment target to prevent bone loss induced by microgravity. Previous studies demonstrated that miR-139-3p was associated with several types of cancer. In colorectal cancer patient serum and cancer tissues, miR-139-3p was silenced^37^. In addition, miR-139-3p was decreased in cervical cancer tissues and cell lines. MiR-139-3p could inhibit cervical cancer cell processes, such as proliferation, migration, and invasion. Moreover, miR-139-3p could downregulate NOB1 expression to induce cell apoptosis^38^. Downregulating miR-139-3p and miR-139-5p could also enhance the migration and invasion of bladder cancer cells^39^. MiR-139-5p, which originates from opposite arms of the same miRNA precursor for miR-139-3p, could target the Wnt/β-catenin signaling pathway to repress osteogenesis in mesenchymal stem cells^40^. These data indicated that both the 5′- and 3′-strands of pre-miR-139 are important in osteogenesis because they control the activities of different targeting genes. However, the effect of miR-139-3p on osteoblast function has seldom been evaluated, especially in a simulated microgravity environment. Our study further identified that miR-139-3p inhibited osteoblast differentiation and induced osteoblast apoptosis by targeting ELK1.

ELK1, a transcription factor of the ETS family, is an essential component of the mitogen regulation pathway that activates the mitogen-activated protein kinase cascade^41^. ELK1 regulates various cell processes, including cell proliferation and apoptosis, and can be regulated by miRNAs^42^. ELK1 and pELK-1 expression have been positively associated with several types of cancer, including breast cancer, colonic adenocarcinoma, and prostate cancer^43–45^. MiR-150 could regulate ELK1 expression, and ELK1 knockdown could abolish the anti-apoptotic effect of the inhibitor miR-150 in endothelial cells^42^. Increased levels of ELK1 and pELK, which are molecular components of the MAPK signaling pathway, were detected in osteogenesis and are related to osteogenic gene expression^32–35^. Moreover, ELK1 may be involved in Runx2 gene transcription during the progression of osteogenesis^46^. Our research demonstrated that ELK1 was a direct target molecule for miR-139-3p. miR-139-3p overexpression repressed ELK1 and pELK-1 protein expression, whereas miR-139-3p knockdown promoted ELK1 and pELK-1 protein expression in MC3T3-E1 cells. Moreover, there were no remarkable differences in ELK1 phosphorylation levels among the different groups, which indicated that miR-139-3p regulated mainly the total ELK1 protein levels. Our study further proved that ELK1 could promote osteoblast differentiation and inhibit osteoblast apoptosis.

Furthermore, we demonstrated the reciprocal regulation between miR-139-3p and lncRNA ODSM. It has been proven that lncRNAs containing miRNA binding sites could serve as molecular sponges to inhibit miRNA function^27^. However, there are few studies regarding alterations in lncRNA expression in vivo or in vitro under microgravity. To investigate the effect of lncRNAs on osteoblast function under microgravity, microarray expression profiling of lncRNAs was conducted in MC3T3-E1 cells undergoing osteogenic differentiation under simulated microgravity; a total of 857 lncRNAs were altered^36^. Among these altered lncRNAs, lncRNA ODSM was confirmed to interact with miR-139-3p and to be downregulated in MC3T3-E1 cells cultured under simulated microgravity environment. Further evaluation suggested that lncRNA ODSM could enhance osteoblast differentiation. To the best of our knowledge, the function of lncRNA ODSM has not been previously reported.

**Fig. 8:**
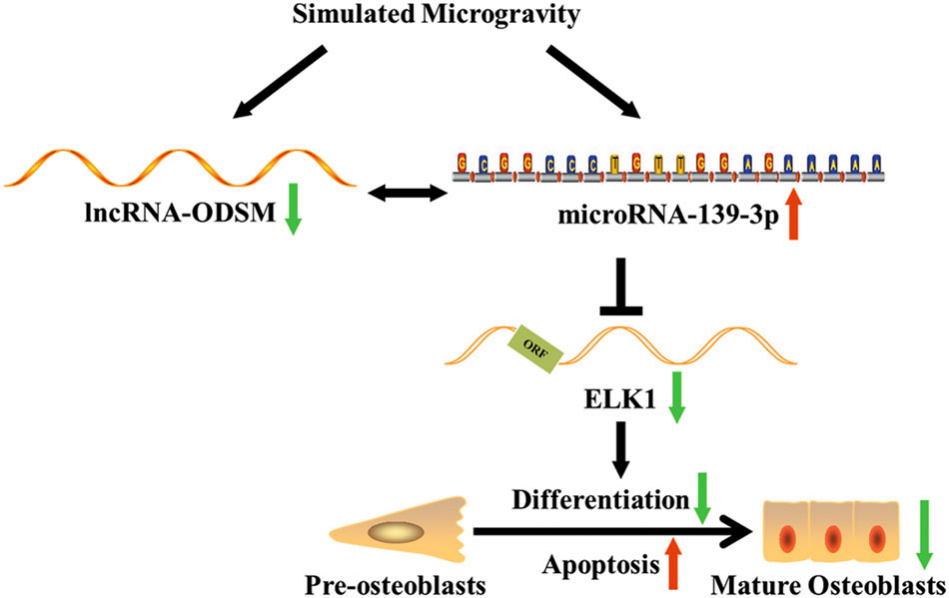
Schematic representation of the lncRNA ODSM/miR-139-3p/ELK1 signaling pathway in osteoblasts. In this mechanism, miR-139-3p is upregulated and interacts with downregulated lncRNA ODSM in osteoblasts under simulated microgravity conditions. As a direct and vital target, ELK1 is inhibited by miR-139-3p and then suppresses osteoblast differentiation and promotes osteoblast apoptosis

In conclusion, this study revealed that miR-139-3p was upregulated in the femurs of hindlimb unloading mice and MC3T3-E1 cells under simulated microgravity. MiR-139-3p inhibited osteoblast differentiation and bone formation and promoted osteoblast apoptosis, which partly depended on ELK1 regulation. ELK1 was confirmed to be directly targeted by miR-139-3p and to regulate MC3T3-E1 cell function. lncRNA ODSM suppressed miR-139-3p expression and enhanced osteoblast differentiation (Fig. 8). Our research revealed the function of the lncRNA ODSM/miR-139-3p/ELK1 signaling pathway in osteoblasts and indicated the promising value of miR-139-3p in the preventative treatment of bone loss induced by microgravity or disuse osteoporosis.

## Materials and methods

### Simulated microgravity

The hindlimb unloading model is a well-accepted animal model of bone loss induced by simulated microgravity. Male C57BL/6J mice aged 6 months were maintained under standard conditions (12 h light, 12 h dark cycle, 21 °C controlled temperature). The mice were suspended by the tail at a 30° angle to the floor with only their forelimbs touching the floor; this allowed the mice to move and access food and water freely. After 3 weeks of tail suspension, the mice were anesthetized. Next, the bilateral femurs and tibiae were removed from the mice. Micro-computed tomography (µCT) and quantitative real-time PCR (qRT-PCR) were used for further analyses. These studies were approved by Air Force Medical University Animal Ethics and Experimental Safety Committee (NO. 14022) and were performed according to the approved guidelines.

2D-clinorotation (developed by China Astronaut Research and Training Center, Beijing) is widely used to simulate a microgravity environment for cells on the ground. This procedure was performed as described previously^24^. Briefly, MC3T3-E1 cells were placed onto coverslips at a density of 1 × 10^5^ cells. After the cells adhered to the wall, the coverslips were inserted into a chamber filled with culture medium and kept 12.5 mm away from the chamber′s rotational axis. Next, the lids of the vessels were screwed down tightly after the bubbles were fully removed. Finally, the chambers were placed into a clinostat and rotated around a horizontal axis at 24 rpm. The vertical rotation groups were used as controls. The clinostat was placed in an incubator at 37 °C.

### μCT analysis

After fixing in 4% paraformaldehyde for 24 h, each mouse femur was scanned by a μCT scanner (Siemens, Germany) at an energy of 80 kV and 500 mA. Each femur was scanned over a total angle of 360° at incremental angles of 0.5°. The scanning time was 800 ms/frame with a resolution of 10.44 μm. A region of interest (ROI) was chosen to represent the microstructure of the femur. The ROI was 15 μm above the proximal epiphyseal growth plate and selected as a 2.5 × 2.5 × 3 mm^3^ cube. The indices, including BMD, BV/TV, Tb.Th, Tb.N, Tb.Sp, and TbPF, were analyzed by COBRA software for μCT. These data were collected for blinded analyses. The statistical results were presented according to Weissgerber et al.^47^.

### Histology

The femurs were removed and decalcified in a decalcifying solution with EDTA (Beyotime Biotechnology, Shanghai, China). Then, the femurs were dehydrated, embedded in paraffin, cut, and stained according to the manufacturer’s protocol (Sigma Aldrich, USA) for Masson and ALP activity staining analyses. The statistical results were presented according to Weissgerber et al.^47^.

### TUNEL assay

The DeadEnd™ Fluorometric TUNEL System (Promega, USA) was used to conduct TUNEL staining analyses. Apoptotic cells in the femoral segment were detected from five perspectives. The ratios of TUNEL-positive nuclei were analyzed using Olympus cellSens Standard software. The statistical results were presented according to Weissgerber et al.^47^.

### qRT-PCR analysis

Using RNAiso Plus (TaKaRa, Japan), total RNA was extracted from bone tissue or cells. For the quantification of miRNA, a Mir-X miRNA First-Strand Synthesis Kit (TaKaRa, Japan) was used to prepare the cDNA. SYBR^®^ Premix Ex Taq^TM^ II (TaKaRa, Japan) was used to quantitatively detect target gene expression. U6 small nuclear RNA was used as a loading control. For the quantification of mRNA, cDNA was synthesized using a PrimeScript^®^ RT Master Mix reagent kit (TaKaRa, Japan). The subsequent real-time PCR detection was conducted using SYBR^®^ Premix Ex Taq^TM^ II (TaKaRa, Japan) and a CFX96 real-time PCR detection system (BIO-RAD, USA). GAPDH was used as a reference gene. The primers used for real-time PCR are listed in Supplementary Table 1.

### Cell culture

The mouse pre-osteoblast MC3T3-E1 cell line was obtained from the Cell Bank of the Chinese Academy of Sciences (Shanghai, China) and cultured in DMEM (HyClone) supplemented with 10% FBS (HyClone) and 1% penicillin/streptomycin (HyClone) under standard cell culture conditions of 5% CO_2_, 95% humidity, and 37 °C. Passages 8–12 of the cells were used for the experiments. To conduct functional experiments on osteogenic differentiation, MC3T3-E1 cells were induced with osteogenic medium supplemented with 100 nM dexamethasone, 10 mM β-glycerophosphate (Sigma), and 50 μM ascorbic acid.

### Cell transfection

The miRNA regulators were transfected into cells using Lipofectamine 2000 (Invitrogen, USA). The concentration of miR-139-3p mimic and its negative control (RiboBio, China) for transfection was 50 nM, and the concentration of the inhibitor and its negative control (RiboBio, China) for transfection was 100 nM. The ELK1 (pEX-ELK1) and lncRNA ODSM (pEX-ODSM) vectors were purchased from GenePharma (Shanghai, China). Additionally, Supplementary Table 1 shows the sequences of the siRNAs and negative controls for ELK1 and lncRNA ODSM. The siRNA concentration for transfection was 80 nM, and the plasmid concentration for transfection was 200 ng/μl. The siRNAs and plasmids were transfected into cells using Lipofectamine 3000 (Invitrogen, USA).

### Western blotting analysis

MC3T3-E1 cells were harvested into RIPA buffer (Thermo Scientific, USA). Equal amounts of protein samples were loaded onto NuPage Bis-Tris polyacrylamide gels (Invitrogen, USA). Next, the proteins were transferred onto polyvinylidene difluoride membranes. These membranes were then blocked with 5% milk for 4 h at room temperature and subsequently incubated at 4 °C overnight with primary antibodies for the following specific genes: Runx2 (1:1000; Cell Signaling Technology, USA), Bglap (1:500; Abcam, USA), Col1a1 (1:1000; Abcam, USA), ELK1 (1:500; Abcam, USA), pELK1 (1:1000; Abcam, USA), Bax (1:1000; Cell Signaling Technology, USA), caspase-3 (1:1000; Cell Signaling Technology, USA), Bcl-2 (1:1000; Cell Signaling Technology, USA), and GAPDH (1:5000; Proteintech, USA). Next, the membranes were incubated with a peroxidase-conjugated secondary antibody (1:5000; Jackson, USA), and the signals were visualized using Super Signal West substrate (Thermo Fisher Scientific, USA). Densitometry analyses were performed using ImageJ software (NIH).

### Alkaline phosphatase activity assay

After rinsing with PBS, MC3T3-E1 cells were harvested with mammalian protein extraction reagent (Pierce, USA). Then, the supernatants were collected after 12,000 × *g* centrifugation for 15 min. An ALP assay kit (Nanjing Jiancheng Technological Inc., China) was used to measure ALP activity in the supernatants. A BCA protein assay kit (Thermo Fisher Scientific, USA) was used to quantify the cellular protein concentration. ALP activity (IU/L) was set as the production of 1 nmol p-nitrophenol from 1 μg of total cellular protein in 1 min.

### Alkaline phosphatase staining

After induction in osteogenic medium for 7 days, MC3T3-E1 cells were subjected to ALP staining using an NBT/BCIP staining kit (Beyotime Biotechnology, China) according to the manufacturer’s protocol. Staining for each group was replicated three times, and a digital camera recorded representative images.

### Flow cytometry

Trypsin digestion was performed on MC3T3-E1 cells with a 0.125% trypsin solution. Then, the cells were washed with PBS and centrifuged at 1000 rpm for 5 min. After resuspension in PBS, the cells were stained using an Annexin V-FITC Apoptosis Detection Kit (BioVision, USA). Next, the apoptosis rates were analyzed by flow cytometry (BD Bioscience, USA).

### Hoechst staining

A Hoechst staining kit (Beyotime Biotechnology, China) was used to distinguish apoptosis in MC3T3-E1 cells according to the manufacturer’s instructions. Briefly, after fixation in 4% paraformaldehyde, the cells were stained with a Hoechst 33258 staining solution. Then, the fluorescence intensity of the stained cells was measured with an Olympus fluorescent microscope (Olympus Corporation, Japan).

### Luciferase assay

293T cells with low endogenous miRNA expression were selected. Then, the wild-type ELK1 3′UTR sequence (WT) or an ELK1 3′UTR mutant sequence (MUT) of the miR-139-3p binding site was generated. Next, the sequences were inserted into the vector with PmirGLO dual-luciferase miRNA target expression (Promega, USA). Similarly, the fragment of lncRNA ODSM containing the predicted miR-139-3p binding site or a corresponding mutated sequence was fused to the vector with PmirGLO dual-luciferase miRNA target expression. Then, the 293T cells were co-transfected with miR-139-3p (mimic, inhibitor or their negative controls) and the ELK1 or lncRNA ODSM vector (WT or MUT) using Lipofectamine 2000 (Invitrogen). Finally, firefly and Renilla luciferase activities were detected using the dual-luciferase assay system (Promega, USA).

### Immunofluorescence

After rinsing with cold PBS, MC3T3-E1 cells were immobilized with 4% paraformaldehyde for 15 min and permeated with 0.025% Triton X-100 for 10 min. Then, the cells were incubated with 1% normal goat serum for 1 h and with a primary ELK1 antibody (1:100; Abcam, USA) overnight at 2–8 °C. Next, the cells were rinsed three times and incubated with secondary antibody conjugated to FITC (Abcam, USA) for 1 h. Finally, the cells were stained with DAPI for 10 min at room temperature, and fluorescence images were taken using an FV1000 confocal microscope (Olympus, Japan).

### RNA-FISH

RNA fluorescence in situ hybridization (RNA-FISH) was used to detect the localization of lncRNA ODSM in MC3T3-E1 cells. After immobilization with 4% paraformaldehyde for 20 min at room temperature, the cells were prehybridized with a hybridization solution. Then, the cells were incubated with the lncRNA ODSM probes overnight at 37 °C. Finally, DAPI was used to stain the nuclei, and fluorescence images were taken using an FV1000 confocal microscope (Olympus, Japan).

### Anti-Ago2 immunoprecipitation

An anti-Ago2 immunoprecipitation assay was conducted using an RNA binding protein immunoprecipitation kit (MilliporeSigma, USA) with an anti-Ago2 antibody (Abcam, USA). A normal mouse IgG was used as a negative control. The PrimeScript^®^ RT Master Mix reagent kit (TaKaRa, Japan) was used to conduct qRT-PCR analyses according to the manufacturer’s instructions.

### Statistical analysis

All statistical analyses were performed using SPSS 22.0 software. All numerical data are shown as the mean ± SD from at least three or five duplicate experiments. Statistical significance was tested by two-tailed *t* test or one-way ANOVA. A *P* value < 0.05 was considered to be significant.

## Electronic supplementary material

Supplemental information V
