# Supplementary material for: MicroRNA-139-3p regulates osteoblast differentiation and apoptosis by targeting ELK1 and interacting with long noncoding RNA ODSM
Source: Cell Death Dis. 2018 Oct 31;9(11):1107. doi: 10.1038/s41419-018-1153-1 (PMC6208413; doi:10.1038/s41419-018-1153-1)
Supplement: Supplementary file 1 — Supplemental information V [file 41419_2018_1153_MOESM1_ESM.docx]

**Supplemental information**

**Supplement MicroRNA-139-3p regulates osteoblast differentiation and apoptosis by targeting ELK1 and interacting with long noncoding RNA ODSM.**

Yixuan Wang, Ke Wang, Zebing Hu, Hua Zhou, Lijun Zhang, Han Wang, Gaozhi Li, Shu Zhang, Xinsheng Cao, Fei Shi

The Key Laboratory of Aerospace Medicine, Ministry of Education, Air Force Medical University, 710032, Xi'an, Shaanxi, China.

**Supplement Table 1. The sequence of primers and siRNAs.**

| Name | Sequence (5'-3') |
| --- | --- |
| miR-139-3p | GCG GCC CTG TTG GAG AAA AA |
| miR-339-3p | GCG ACA GAG CCG AAA AAA AAA |
| miR-19a-3p | CAA ATC TAT GCA AAA CTG AAA AAA |
| miR-487b-3p | ATC GTA CAG GGT CAT CCA CTT |
| miR-34b-5p | GGA GGC AGT GTA ATT AGC TGA |
| Runx2-F | GAA CCA AGA AGG CAC AGA CAG A |
| Runx2-R | GGC GGG ACA CCT ACT CTC ATA C |
| ALP-F | GCA GTA TGA ATT GAA TCG GAA CAA C |
| ALP-R | ATG GCC TGG TCC ATC TCC AC |
| Bglap-F | GAC CGC CTA CAA ACG CAT CTA |
| Bglap-R | CAG AGA GAG AGG ACA GGG AGG A |
| Col1a1-F | GAC ATG TTC AGC TTT GTG GAC CTC |
| Col1a1-R | GGG ACC CTT AGG CCA TTG TGT A |
| ELK1-F | CGG GCC TTG CGG TAC TAC TAT G |
| ELK1-R | TCA GTG AG CAC CCT GCA AC |
| LncRNA ODSM-F | GCA AAG TTG TGC CAT CCA G |
| LncRNA ODSM-R | CCA CTT AGC GAT AAA AAG AAA TCT |
| GAPDH-F | TGT CCG TCG TGG ATC TGA |
| GAPDH-R | TTG CTG TTG AAG TCG CAG GAG |
| siR-ELK1 sense | GCC AGA AGU UUG UCU ACA ATT |
| siR-ELK1 antisense | UUG UAG ACA AAC UUC UGG CTT |
| siR-ODSM sense | GCU CUC UCC CUG ACU GUU ATT |
| siR-ODSM antisense | UAA CAG UCA GGG AGA GAG CTT |
| siR-NC sense | UUC UCC GAA CGU GUC ACG UTT |
| siR-NC antisense | ACG UGA CAC GUU CGG AGA ATT |

**Supplement Table 2. Prediction of miR-139-3p target genes using miRanda, miRDB and TargetScan.**

**2.1 Prediction of miR-139-3p target genes using miRanda**

| Target Rank | Gene | miSVR score |
| --- | --- | --- |
| 1 | Elk1 | -1.67 |
| 2 | Rbm26 | -1.31 |
| 3 | Plagl1 | -0.34 |

**2.2 Prediction of miR-139-3p target genes using miRDB**

| Target Rank | Gene | Target Score |
| --- | --- | --- |
| 1 | Elk1 | 98 |
| 2 | 6430590A07Rik | 82 |
| 3 | Thap11 | 79 |

**2.3 Prediction of miR-139-3p target genes using TargetScan**

| Target Rank | Gene | Cumulative weighted context++ score |
| --- | --- | --- |
| 1 | Cul4a | -1.34 |
| 2 | Lrrc61 | -1.33 |
| 3 | Pnkd | -1.33 |
| 21 | Elk1 | -0.65 |

**2.4 Prediction of miR-139-3p target genes among down-regulated lncRNAs under simulated microgravity using miRanda**

| Target Rank | Gene | Score |
| --- | --- | --- |
| 1 | NONMMUT002009 (lncRNA ODSM) | 161 |
| 2 | NONMMUT055168 | 150 |
| 3 | NONMMUT002090 | 147 |
| 4 | NONMMUT020642 | 143 |
| 5 | NONMMUT048960 | 143 |

**
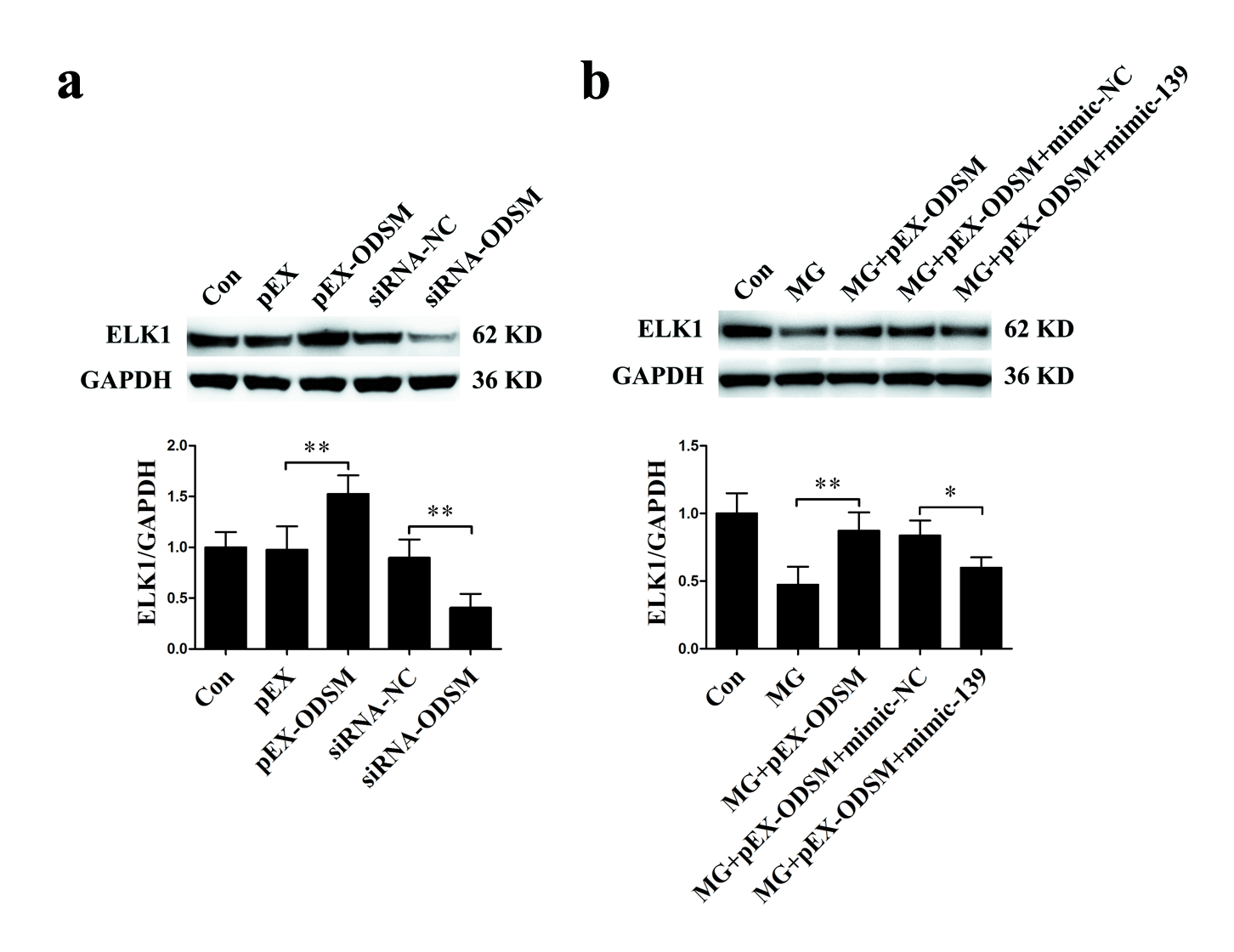
Supplementary Fig 1**

**Supplementary Fig 1.** **LncRNA ODSM regulates ELK1 expression and overexpression of miR-139-3p partly reverses ELK1 level induced by lncRNA ODSM under simulated microgravity.** (a) pEX-ODSM, siRNA-ODSM and their negative controls were transfected into MC3T3-E1 cells. Protein levels of ELK1 in osteoblasts (*N*=3) (b) pEX-ODSM and mimic-139 or its negative control were co-transfected into MC3T3-E1 cells and osteoblast were cultured under simulated microgravity for 48 h. Protein levels of ELK1 in osteoblasts (*N*=3) **P <* 0.05, ***P <* 0.01 vs. control.
